# Complementary approaches to the study of decision making across the adult life span

**DOI:** 10.3389/fnins.2013.00243

**Published:** 2013-12-20

**Authors:** Gregory R. Samanez-Larkin, Shu-Chen Li, K. Richard Ridderinkhof

**Affiliations:** ^1^Department of Psychology, Yale UniversityNew Haven, CT, USA; ^2^Lifespan Developmental Neuroscience, Department of PsychologyTU Dresden, Dresden, Germany; ^3^Center for Lifespan Psychology, Max Planck Institute for Human DevelopmentBerlin, Germany; ^4^Department of Psychology, University of AmsterdamAmsterdam, Netherlands; ^5^Amsterdam Brain and Cognition, University of AmsterdamAmsterdam, Netherlands

**Keywords:** aging, reward, risk, decision making, intertemporal choice, learning, memory, computational modeling

Learning to choose adaptively between different behavioral options in order to reach goals is a pervasive task in life for people of all ages. Individuals are often confronted with complex, uncertain situations that nonetheless require decisive actions that would facilitate the pursuit of short-term or long-term goals. Adaptive decision making as such entails interactions between processes that monitor the choice-outcome relations as well as processes that evaluate these relations with respect to goal relevance. These dynamics implicate close interplays between attention, learning, memory, motivation, and emotion, which are subserved by cortical-subcortical networks and are neurochemically regulated by transmitters, such as norepinephrine, dopamine, and serotonin. Across the life span, these functional brain circuits as well as neurotransmitter systems undergo basic biological maturation and senescence as well as plasticity due to the accumulation of experience or changes in motivational goals (Braver and Barch, 2002; Li and Sikström, 2002; Düzel et al., 2010; Li et al., 2010; Mohr et al., 2010; Li, 2013). Studying decision making across different adult life periods may shed light on how the very processes of decision making adapt to constraints on brain resources due to aging, how these processes benefit from experience, or how decision making is influenced by shifting goals.

Multidisciplinary research on decision making and aging is growing at a rapid pace (Brown and Ridderinkhof, 2009; Eppinger et al., 2011; Samanez-Larkin, 2011; Hämmerer and Eppinger, 2012; Samanez-Larkin and Knutson, 2014). Given the increasing interest in work in this area, the aim of this research topic in Frontiers in Neuroscience is to open a forum for the subfield of decision science that focuses on comparing and contrasting decision making in people of different ages. In this series, we have highlighted the range of complementary methodological approaches that are currently being used.

Here we feature empirical work ranging from behavioral (Mather and Schoeke, 2011; Cavanagh et al., 2012; Shivapour et al., 2012; Spaniol and Wegier, 2012; Westbrook et al., 2012; Worthy and Maddox, 2012) and computational (Samanez-Larkin et al., 2011a; Cavanagh et al., 2012; Worthy and Maddox, 2012) to cognitive neuroscience (Samanez-Larkin et al., 2011a) and non-human animal research (Gilbert et al., 2011), investigating age similarities and differences in decision making, together with theoretical perspectives (Mata et al., 2012) that integrate existing evidence and provides new insights. The papers also cover a broad range of topics including reward effects on learning and memory, risky decision making, intertemporal choice, strategy use, and financial decision making in healthy adults (Gilbert et al., 2011; Mather and Schoeke, 2011; Samanez-Larkin et al., 2011a; Cavanagh et al., 2012; Mata et al., 2012; Shivapour et al., 2012; Spaniol and Wegier, 2012; Westbrook et al., 2012; Worthy and Maddox, 2012) along with a complementary study on susceptibility to misleading advertisements in individuals with frontal cortical damage (Asp et al., 2012).

Despite stereotypes of old age as a life period characterized by global cognitive declines, there are many decision-related processes that remain intact or might even be enhanced in older adults. In this Research Topic, Mather and Schoeke (2011) and Cavanagh et al. (2012) provide evidence for reward effects on memory and risky decision making, respectively, in younger and older adults. In the past several years a growing literature on learning and decision making provides contradictory evidence for differential sensitivity to monetary rewards relative to losses across adulthood (Hämmerer and Eppinger, 2012; Samanez-Larkin and Knutson, 2014). These opposing findings stand in contrast to a larger, related, and more consistent literature on valence effects in attention and memory. These studies find that older adults pay increasingly more attention to and better remember positive relative to negative material (Carstensen and Mikels, 2005; Mather and Carstensen, 2005; Carstensen, 2006). The study by Mather and Schoeke (2011) lies at the intersection of these sets of findings by examining the impact of associating monetary gains and losses with pictures, and subsequently testing memory for these pictures. They observe similar effects in both age groups: Both younger and older adults better remember pictures that were associated with positive outcomes (monetary gains or loss avoidance) than negative outcomes (missed gains or realized losses). There were no age differences; both younger and older adults showed the same reward-enhancement of memory suggesting that the motivating effects of monetary rewards might be preserved across adulthood (Castel et al., 2011). Using a computational model of decision making in the BART task, Cavanagh et al. (2012) show that older adults are not only as sensitive to reward as younger adults but that they may be even more sensitive to reward compared to young adults in some situations. However, in this task the reward effects lead to excessively risky behavior that may be detrimental to performance in some contexts.

The perspective piece by Mata et al. (2012) in this series provides an in-depth discussion of adaptation to different decision contexts suggesting that what may be an effective strategy in some contexts may be maladaptive in others. An example of this is highlighted in the paper by Worthy and Maddox (2012) that uses computational models of learning and decision making to identify strategy differences between younger and older adults. Consistent with previous studies of age differences in strategy use (Mata et al., 2007, 2010), Worthy and Maddox (2012) show that older adults use a simpler strategy (win-stay/lose-shift) compared to younger adults (who are better fit by a more traditional reinforcement learning model). Using two different decision environments, these authors show that such simpler strategies are adaptive in one context (where future performance is dependent on current choice behavior) but maladaptive in the other context (where future performance is independent of current choice behavior). Related to the issue of adaptation across contexts, Spaniol and Wegier (2012) in this series provide evidence that although older adults show reduced information search in a risky decision task compared to younger adults. Consistent with prior work (Mata and Nunes, 2010), both younger and older adults shift their information search strategies according to the relative probability of monetary gains providing evidence for some level of intact adaptation across adulthood.

Given the evidence for the adaptability of choice in old age, these findings might suggest that it would be beneficial to provide decision strategies to older adults in scenarios where they would be most vulnerable to making mistakes (Samanez-Larkin et al., 2011b). Westbrook et al. (2012) in this series attempt to do just that. They trained younger and older adults to use a specific strategy in a risky decision task to reduce excessive risk aversion. They found no age differences in risk aversion in the task at baseline, but they did observe differential effectiveness of the strategy training across age groups. The older adults tended to abandon the strategy over time and, as a result, the effect of strategy training was smaller overall in the older compared to younger adults. The authors suggest that the effects may be related to goal neglect deficits such that older adults have difficulty maintaining the strategy. However, it is also possible that they perceive the strategy to be less effective than their own baseline strategy and hence intentionally utilize it less and less over time. Independent of whether the age differences are due to cognitive constraints or personal preferences, the study suggests that instructional training or the encouragement of a specific strategy may be less effective in older adults.

Two of the papers in this series examine age differences in discounting of temporal delays (Samanez-Larkin et al., 2011a) and probability (Gilbert et al., 2011). A growing body of prior research has shown that older adults are often more likely to wait for larger, temporally delayed rewards (Löckenhoff, 2011). For the relatively short time delays (seconds to weeks) used in these studies, older adults show reduced discounting of time delays. Samanez-Larkin et al. (2011a,b) in this series provide evidence for similar ventral striatal sensitivity to non-delayed and delayed rewards suggesting that there is a lower temporal discount rate in striatal brain activity in old age. The enhanced sensitivity to immediacy in young adults seems to be reduced across adulthood. This age by delay interaction in the striatum subsequently replicated in another fMRI study with humans (Eppinger et al., 2012) and the same pattern was observed in the orbitofrontal cortex of older compared to younger rodents (Roesch et al., 2012). This increased delay cost tolerance with age may be viewed as adaptive in that larger rewards are obtained after longer delays, however, for some individuals a more general reduction in cost integration may lead to excessively risky choice behavior. Gilbert et al. (2011) in this series show that younger and older animals make similar choices in a probabilistic choice task overall, but that there are strong individual differences in the older animals. A subset of older rats showed a reduced sensitivity to probability, maintaining a preference for probabilistic over certain rewards even when the expected values were lower. These same animals showed a reduced sensitivity to time delays compared to younger animals in a prior study (Simon et al., 2010), demonstrating that there are a subset of older animals who may be overly focused on reward magnitude and are not integrating potential benefits with costs.

Older adults are not always more willing to wait. Shivapour et al. (2012) in this series show that older adults are more likely to make financial decisions without much deliberation compared to younger adults who are more likely to put off financial decisions for later. Although older adults showed higher levels of financial knowledge and reported being highly motivated to prevent financial losses (Shivapour et al., 2012), older individuals are often targets of financial fraud attempts. It is not clear whether they are more susceptible to fraud, but they are disproportionately targeted likely due to their greater financial assets compared to young adults. The age-related positivity effect in attention and memory mentioned above may be beneficial for the promotion of well being in everyday life (Carstensen et al., 2000, 2011), but to the extent that these affective biases are domain general they may also have negative consequences for some financial decisions. Recent evidence for reduced sensitivity in the anterior insula in older age to the prospect of financial loss (Samanez-Larkin et al., 2007), unfair offers in social decision making tasks (Harlé and Sanfey, 2012), and untrustworthy faces (Castle et al., 2012) suggests that older adults may be more vulnerable to making financial mistakes such as falling victim to fraudulent investments. Normal aging is characterized by gradual structural decline of the prefrontal cortex (Grady, 2012), but there are large individual differences in the rate of this decline. To test an extreme case of loss of frontal cortical systems, Asp et al. (2012) in this series show that individuals with frank damage to the prefrontal cortex were more influenced by misleading advertisements. The study suggests that individuals with steeper rates of frontal cortical decline may be the most vulnerable to making financial mistakes.

Overall the series of papers identifies areas of potential improvement, preservation, and decline in decision-related processes across adulthood. A potentially interesting area that we did not cover in this series but one that is gaining recent attention is the examination of differential effects of genetic variability on decision making across the life span (Hämmerer et al., 2013). This collection of papers highlights the range of approaches being used in this area, and the set together provides promising evidence that future discoveries and refinements of theoretical models of human aging will be more comprehensive through the increasing adoption of a multi-method, multidisciplinary approach.
